# Validation of the point-of-care (POC) technologies Xpert HIV-1 Qual and m-PIMA HIV 1/2 detect for early diagnosis of HIV-1 and HIV-2 in Senegal

**DOI:** 10.11604/pamj.2022.43.42.32714

**Published:** 2022-09-27

**Authors:** Aissatou Sow Ndoye, Halimatou Diop Ndiaye, Marième Diallo, Aminata Diack, Khadydiatou Coulibaly, Gora Lo, Brianan Kiernan, Pauline Yacine Sène, Ousseynou Ndiaye, Ndèye Fatou Ngom, Mengue Fall, Karim Diop, Sokhna Bousso Gueye, Assane Dieng, Charlotte Lejeune, Cheikh Tidiane Ndour, Coumba Toure Kane

**Affiliations:** 1 Laboratoire Bactériologie-Virologie, Hopital Aristide Le Dantec, Dakar, Senegal,; 2 Institute for Health Research, Epidemiological Surveillance and Training, Dakar, Senegal,; 3 Clinton Health Access Initiative, Dakar, Senegal,; 4 Hopital d´Enfants Albert Royer, Dakar, Senegal,; 5 Division de Lutte Contre le SIDA/IST, Dakar, Senegal,; 6 United Nations International Children's Emergency Fund (UNICEF), Dakar, Senegal

**Keywords:** HIV, early infant diagnosis, infant, evaluation, performance

## Abstract

**Introduction:**

early infant diagnosis (EID) is crucial in the prevention of mother to child transmission (PMTCT) of human immunodeficiency virus (HIV) and is an essential component for the elimination of HIV. EID can be strengthened in resource-limited countries by the introduction and the roll out of real-time polymerase chain reaction (PCR) technologies via point-of-care (POC) devices which improves treatment in remote areas and reduces turnaround time for clinicians and patients to receive results and linkage to care. The objective of this study was to evaluate the performance of Xpert® HIV-1 Qual Assay (Cepheid) and m-PIMA™ HIV 1/2 Detect (ABBOTT) for EID of HIV-1 and HIV-2.

**Methods:**

the performance of the Xpert® HIV-1 qual device was evaluated with 192 samples including 100 dried blood spot (DBS) samples from the National Reference Laboratory biobank (71 negative and 29 positive samples) and an additional 92 whole blood samples collected from infants from neonatal departments. These infants from seven treatment centers in the Dakar region were born to mothers infected with HIV-1 (n=91), HIV-2 (n= 8) or HIV-1/2 (n=1). The m-PIMA™ HIV 1/2 detect assay was evaluated on whole blood samples (n=100) with 92 HIV-1 samples and 8 HIV-2 samples from children born to HIV-infected mothers. The Cobas AmpliPreP/Cobas TaqMan (CAP/CTM) platform from Roche Diagnostic Laboratories was used as a reference for HIV-1 diagnosis and the Generic HIV-2 Viral Load Assay (Biocentric) was used as a reference for HIV-2 diagnosis. Performance was evaluated by calculating sensitivity (Se), specificity (Sp), positive predictive value (PPV), negative predictive value (NPV) and Cohen's kappa coefficient.

**Results:**

for HIV-1 detection on GeneXpert and m-PIMA, no discordance was found on the samples tested, i.e. a sensitivity of 100% (95% CI: 93.9-100%), a specificity of 100% (95% CI: 97.5-100%), a positive predictive value (PPV) of 100% (95% CI: 93.9-100%) and a negative predictive value (NPV) of 100% (95% CI: 97.5-100%). Agreement with Cobas AmpliPrep/Cobas TaqMan (CAP/CTM) was 100% with a Kappa coefficient of 1 (p<0.001, 95% CI) for both techniques. Similarly, the comparison between m-PIMA and generic biocentric for the detection of HIV-2 on the 8 samples tested showed perfect agreement.

**Conclusion:**

these results confirm the excellent performance of the Xpert® HIV-1 qual and m-PIMA™ HIV1/2 detect tests for the detection of HIV-1 and HIV-2 and encourage the extension of POC tests to improve access to EID in Senegal.

## Introduction

Pediatric HIV infection remains a major public health problem with 1.8 million HIV-infected children worldwide, including 1.6 million HIV infections in sub-Saharan Africa and 95,000 (60,000-00,000) HIV-related deaths in 2019 [1]. These infections result from mother-to-child transmission (MTCT) of HIV during pregnancy, childbirth or breastfeeding [2]. The goal of eliminating MTCT is an integral part of the overall global HIV strategy and includes different interventions including screening of pregnant women and early HIV testing in newborns [3,4]. Early infant diagnosis (EID) is a key factor in the prevention of mother-to-child transmission (PMTCT) and medical management of children. Early diagnosis is critical to know the HIV status of infants and to improve prevention and treatment interventions as peak mortality occurs between the ages of six weeks to four months for children with HIV infection [5]. This life saving diagnosis should be routinely performed in all infants born to HIV-positive mothers under 6 weeks of age or at birth in women at high risk of transmission [4,6-8]. Due to detectable maternal antibodies in newborns, this diagnosis must be performed with molecular diagnostic techniques which are not always accessible to populations living in rural areas [9]. In 2016, only 20% of HIV-exposed children received EID testing in the first two months post-birth in West and Central Africa [10] and in 2020 only one third (33%) of HIV-exposed children were tested in their first two months post-birth [1].

In contrast, in Southern Africa, significant progress has been made in early diagnosis through the introduction of alternative technologies, notably point-of-care (POC) tests, which have made HIV diagnosis in newborns more accessible. These tests, which appear to be a complementary strategy to the existing conventional laboratory system, have significantly reduced the turnaround time to deliver PCR test results, to manage patients, and the risk of losing patients during follow-up visits [7,11]. Among these alternative techniques, two POC assays are prequalified by WHO for EID namely, m-PIMA™ HIV-1/2 Detect (Abbott Technologies GmbH) and Xpert® HIV-1 Qual Assay (Cepheid AB) [11].

Senegal, like other countries in the West and Central Africa Region, is committed to the elimination of MTCT but many challenges remain. In fact, only 35% and 43% of children born to HIV positive mothers had benefited from early infant diagnosis in 2017 and 2018 respectively. There was therefore an urgent need to introduce POC testing to ensure better coverage of EID; however, the analytical performance of these techniques needs to be validated due to the high genetic diversity of HIV strains circulating in Senegal.

The objective of this study was to evaluate the performance of the POC Xpert® HIV-1 Qual Assay (Cepheid AB) and m-PIMA™ HIV-1/2 Detect (Abbott) tests on whole blood and DBS samples for the neonatal diagnosis of HIV-1 and HIV-2.

## Methods

The Xpert® HIV- 1 Qual Assay (Cepheid) was evaluated for the detection of HIV-1 deoxyribonucleic acid (DNA) from whole blood and/or DBS samples in comparison to the reference technique COBAS® AmpliPrep/COBAS® TaqMan HIV-1 qualitative test version 2.0 (CAP/CTM® version 2) from Roche Laboratories (Roche® Molecular Systems, Inc., Branchburg, NJ). The evaluation of the m-PIMA™ HIV-1/2 Detect Assay (Abbott) was performed only on whole blood samples in comparison to the Cobas TaqMan reference technique from Roche Laboratories and Generic HIV-2® viral load (Biocentric) for detection of HIV-1 and HIV-2 DNA, respectively.

**Sampling:** the evaluation of the Xpert® HIV-1 Qual test included 100 DBS samples obtained as part of routine EID testing at the national HIV reference laboratory in Senegal *(Laboratoire de Bactériologie-Virologie du CHNU Aristide le Dantec)* and included positive and negative DBS specimens after detection by the Roche CAP/CTM test. An additional 100 whole blood samples were collected from infants born to HIV positive mothers at selected HIV treatment centers to prospectively evaluate the Xpert® HIV-1 Qual test for HIV-1 detection and the m-PIMA ™ HIV 1/2 detect test for HIV-1 and HIV-2 detection. Blood samples for HIV-1 DNA testing were used for Xpert and m-PIMA testing and to prepare DBS for comparative testing with CAP-CTM. Blood samples for HIV-2 DNA testing were used to perform the m-PIMA testing and to prepare aliquots of plasma obtained by centrifugation at 2500 rpm for 10 minutes for the biocentric comparative testing on the Applied Biosystems ABI 7300 device. These plasma samples were stored at -80°C until the tests were performed.

**Detection of HIV-1 DNA with the Xpert® HIV-1 Qual Assay:** detection of HIV-1 DNA with the Xpert® HIV-1 Qual Assay kit uses the principle of reverse transcription (RT)-PCR by amplifying the HIV-1 long terminal repeat (LTR) target region. This detection was performed according to the manufacturer's instructions by adding 100 μl of whole blood to the reaction chamber of the GeneXpert cartridge for the prospective study. For the retrospective study, HIV-1 DNA detection was performed according to the manufacturer's recommendations from the eluate of a dried blood. Whether whole blood or eluate, all steps allowing extraction, nucleic acid purification, amplification and simultaneous detection occur in the cartridge and results were obtained after 90 min.

**Detection of HIV-1 and/or HIV-2 DNA with the m-PIMA™ HIV 1/2 detect:** the detection of HIV- 1 or HIV-2 DNA with the m-PIMA assay was performed using the m-PIMA™ HIV-1/2 detect kit (Abbott Diagnostics) consisting of a single-use cartridge within which multiplex PCR takes place. The cartridge contains all the necessary reagents to perform extraction, amplification, and detection from a 25μl whole blood test sample and results are obtained after 58 minutes.

**Reference tests for the diagnosis of HIV-1 and HIV-2:** all samples for HIV-1 DNA were also tested with CAP/CTM V2.0, which is a qualitative test that allows simultaneous detection of HIV-1 pro-viral DNA and an internal control (IC) [12]. This test targets both the HIV-1 *gag* gene and the LTR region, and detection was performed using a dried blood spot made from 70 μl of whole blood. The spot was eluted and agitated under heat (500rpm at 56°C) into a tube containing 1100 μl of COBAS® AmpliPrep/COBAS® TaqMan pre-extraction reagent (SPEX). Subsequently, sample and control preparation were performed in the COBAS AmpliPrep automated HIV-1 DNA extraction system, followed by real-time PCR on the COBAS Taqman 48 analyzer. Plasma samples for HIV-2 detection were tested with the Generic HIV-2 Viral Load Assay (Biocentric) with 500 μL of plasma sample according to manufacturer´s instructions for m-PIMA evaluation.

**Statistical analysis:** diagnostic performance was assessed by calculating sensitivity (Se), specificity (Sp), positive predictive value (PPV) and negative predictive value (NPV). Confidence intervals of 95% were calculated for all analyses. The agreement between the techniques was evaluated by calculating Cohen´s Kappa coefficient which is excellent if K>0.9 [13]. According to WHO, it is recommended that the specificity and sensitivity of molecular diagnostic tests be at least 95% and 98% respectively [14].

**Ethical approval:** the study was approved by the Ethics Committee of the Ministry of Health and Social Action of Senegal under the number 0000093/MSAS/DPERS/CNERS. Written informed consent was obtained from the mothers in the prospective study for EID.

## Results

**Study population:** for the retrospective evaluation, 100 DBS, including 71 DBS with an “undetected” HIV-1 viral DNA result and 29 DBS with a confirmed HIV-1 viral DNA result were included. These DBS were from the 14 regions of Senegal. For the prospective study, 92 children born to HIV-1 infected mothers and 8 to HIV-2 infected mothers were included and a whole blood sample on ethylenediaminetetraacetic (EDTA) tube was collected. These samples were obtained from pediatric hospitals and services in the Dakar Region, Albert Royer Children's Hospital (n=29), Philippe Maguilène Senghor Hospital (n=21), Roi Baudouin Hospital (n=21), Mbao Health Center (n=19), and Youssou Mbargane Public Health Hospital (n=10). Thus, the evaluation included a total of 200 children with a median age of 20 weeks (ranged from 1 to 124 weeks) and 51% of the children were less than 10 weeks old.

Analysis of the 92 prospectively recruited samples using the CAP-CTM reference technique for the qualitative detection of HIV-1 found 1 positive sample, bringing the number of HIV-1 positives to 30 out of the total 200 samples evaluated. Analysis of the remaining 8 samples for qualitative detection of HIV-2 using the Biocentric technique did not find any positive samples.

**Xpert® HIV-1 Qual Assay:** the performance evaluation of the Xpert® HIV-1 Qual test therefore included 190 samples (100 DBS and 90 whole blood) which were tested with 175 valid results and 15 invalid samples, i.e. an invalidity rate of 7.9%. All 30 samples that were positive with the CAP/CTM reference technique were positive with the Xpert® HIV-1 Qual test, i.e. a sensitivity of 100% (95% CI: 93.9-100%). Similarly, all 145 samples that were negative with CAP/CTM were found to be negative with the Xpert^®^ HIV-1 Qual test, i.e. a specificity of 100% (95% CI: 93.9-100%), a PPV of 100% (95% CI: 93.9-100%) and an NPV of 100% (95% CI: 97.5-100%). Agreement was 100% (Table 1) with a Kappa coefficient of 1 (p <0.001, 95% CI) for both techniques.

**Table 1:** comparison of GeneXpert vs CAP/CTM HIV-1 qual for HIV-1 detection

GeneXpert	Cobas AmpliPreP/Cobas TaqMan (CAP/CTM)		
	Positive	Negative	Total
Positive	30	0	30
Negative	0	145	145
Total	30	145	175

**m-PIMA™ HIV ½ Detect test:** the performance evaluation of the m-PIMA™ HIV 1/2 Detect assay included only the 100 samples obtained during prospective recruitment. Out of the 92 whole blood samples tested for HIV-1 viral DNA, 1 was positive and 91 were negative confirming the results of the CAP/CTM, i.e. a sensitivity of 100% (95% CI: 93.9-100%), a specificity of 100% (95% CI: 93.9-100%), a PPV of 100% (95% CI: 93.9-100%) and an NPV of 100% (95% CI: 97.5-100%). Agreement was 100% (Table 2) with a Kappa coefficient of 1 (p<0.001, 95% CI) between the two techniques.

**Table 2:** comparison of m-PIMA qual versus CAP/CTM for the detection of HIV-1 DNA in whole blood

m-PIMA	Cobas AmpliPreP/Cobas TaqMan (CAP/CTM)		
	Positive	Negative	Total
Positive	1	0	1
Negative	0	91	91
Total	1	91	92

For the remaining 8 samples, the HIV-2 DNA test was negative confirming the results of the Biocentric Generic HIV-2 viral load assay with a perfect agreement between the two techniques (Table 3), i.e. a sensitivity of 100% (95% CI: 93.9-100%), a specificity of 100% (95% CI: 93.9-100%), a PPV of 100% (95% CI: 93.9-100%) and a NPV of 100% (95% CI: 97.5-100%). No samples were detected as HIV-2 positive during the prospective study. An error rate of 10.9% with 10 invalid results (errors) was observed, but the tests became conclusive after retesting. Therefore, out of 100 samples tested with the m-PIMA platform for HIV-1 and HIV-2 DNA a perfect match was obtained with an error rate of 10.9%.

**Table 3:** comparison of m-PIMA qual versus generic HIV-2 viral load biocentric for HIV-2 DNA detection

m-PIMA	Generic HIV-2 Biocentric		
	Positive	Negative	Total
Positive	0	0	0
Negative	0	8	8
Total	0	8	8

## Discussion

Acquired immunodeficiency virus (HIV/AIDS) infection remains one of the leading causes of infant and child mortality and morbidity in sub-Saharan Africa. Early diagnosis of HIV infection in children allows for early therapeutic management that limits progression to the AIDS phase [15,16]. POC technologies for HIV represents an excellent opportunity to improve the accessibility of early molecular diagnosis in West Africa, and in Senegal in particular. It is in this context that we conducted this study to evaluate the diagnostic performance of two POC techniques using the Cobas TaqMan test (Roche) for HIV-1 and the Generic HIV-2 viral load (Biocentric) for HIV-2 as references.

A total of 200 samples (100 DBS and 100 whole blood samples) were used in this study. The DBS cards, from routine EID, were only used with the Xpert® HIV-1 Qual test as whole blood samples are required for the m-PIMA technology. The median age of diagnosis in our study population was 20 weeks thus showing a delay in the implementation of diagnosis of children in our study population. Indeed, the median age for EID according to WHO recommendations is 6 weeks, and this was found in studies conducted in South Africa in 2016 on m-PIMA [8] and in Kenya in 2019 on GeneXpert [17]. In this study, the proportion of children who were older than 6 weeks in the retrospective and prospective arm was 74% and 94% respectively.

Performance evaluation of the Xpert® HIV-1 Qual test and m-PIMA™ HIV 1/2 Detect showed 100% sensitivity, 100% specificity for HIV-1 and HIV-2. Comparison results with reference techniques showed perfect agreement with an excellent Kappa coefficient (K=1). A very large body of evaluation work conducted on these POC technologies has shown similar results with respective sensitivities and specificities ranging from 93% to 100% and 99% to 100% with GeneXpert [18-21]. Similarly, the m-PIMA HIV 1/2 technology yielded sensitivities and specificities of 92% to 100% and 95% to 100%, respectively [8,22,23] on specimens from infants under 18 months of age.

However, these performances are variable depending on the age of the children at diagnosis; indeed, sensitivities can go from 90% with m-PIMA™ HIV-1/2 Detect [8,22] to 93% with Xpert® HIV-1 Qual [19] in children at birth to 100% in older children. This reduction in sensitivity in younger children could be explained by the administration of ARVs to the mother in the PMTCT setting.

During the tests, an error rate of 7.9% was noted with the Xpert® HIV-1 Qual which is similar to the error rate obtained in the work of Bwana *et al*. in 2019 (7.65%) [17]. This error rate is higher than the one found in the study conducted Botswana by Ibrahim *et al*. in 2017 [19] and Malawi by Ceffa *et al*. in 2016 [18] where the error rates vary between 2 and 6%; and is also much higher than the error rate obtained in the study by Opollo *et al*. in 2018 (0.7%) [4]. For m-PIMA™ HIV 1/2 Detect, the error rate was 11%. This rate falls within the rates obtained in the studies of Hsiao *et al*. in 2016 in South Africa [8], and Dunning *et al*. in 2017 in Tanzania [22] where the error rate varies between 9 and 15%. The error rates found in this study were mostly related to electrical problems and handling errors by staff who were newly trained [14,18].

For the m-PIMA test, the samples with errors recorded were all re-tested, which were then conclusive, as in most of the studies encountered. For the GeneXpert platform, the re-tests could not be performed due to the limited number of cartridges available. This was a limitation for our study, hence the need to have a sufficient stock with an increase in the number of cartridges to overcome these constraints. Another limitation of the study was the very small number of HIV-2 exposed children included during the entire prospective phase (n=8) and who were compared between m-PIMA HIV 1/2 and Generic Biocentric HIV-2. Although perfect agreement was obtained between the comparison of the techniques with K=1, further work including a much larger sample of HIV-2 infected children is needed to confirm these results.

## Conclusion

Early diagnosis of HIV is critical to the elimination of mother-to-child transmission. The importance of this technology lies in the possibility of early management and a reduction in morbidity and mortality. In this study, no discordance was noted between POC techniques and the references. These satisfactory results reinforce the recommendation to decision-makers and service providers for the introduction and scale up of POC testing. By making EID accessible through the decentralization of POC technologies, this could improve PMTCT across Senegal.

**Funding:** this study was supported by CHAI/UNITAID (Clinton Health Access Initiative).

### What is known about this topic

- Early diagnosis of HIV essential for the elimination of the pandemic, is carried out in reference laboratories with equipment with a long turner around time

### What this study adds

- Extension of POC techniques to improve access to early diagnosis of infants in decentralized areas in Senegal.
